# Italian adaptation of the Edinburgh Social Cognition Test (ESCoT): A new tool for the assessment of theory of mind and social norm understanding

**DOI:** 10.3389/fpsyg.2022.971187

**Published:** 2022-10-28

**Authors:** Sara Isernia, Sarah E. MacPherson, R. Asaad Baksh, Niels Bergsland, Antonella Marchetti, Francesca Baglio, Davide Massaro

**Affiliations:** ^1^IRCCS Don Carlo Gnocchi Foundation ONLUS, Milan, Italy; ^2^Department of Psychology, School of Philosophy, Psychology and Language Sciences, University of Edinburgh, Edinburgh, United Kingdom; ^3^Department of Forensic and Neurodevelopmental Sciences, Institute of Psychiatry, Psychology, and Neuroscience, King’s College London, London, United Kingdom; ^4^Research Unit on Theory of Mind, Department of Psychology, Università Cattolica del Sacro Cuore, Milan, Italy

**Keywords:** social cognition, theory of mind, assessment, social norms, ecological, evaluation

## Abstract

The relevance of social cognition assessment has been formally described in the Diagnostic and Statistical Manual of Mental Disorders-5. However, social cognition tools evaluating different socio-cognitive components for Italian-speaking populations are lacking. The Edinburgh Social Cognition Test (ESCoT) is a new social cognition measure that uses animations of everyday social interactions to assess (i) cognitive theory of mind, (ii) affective theory of mind, (iii) interpersonal social norm understanding, and (iv) intrapersonal social norm understanding. Previous studies have shown that the ESCoT is a sensitive measure of social cognition in healthy and clinical populations in the United Kingdom. This work aimed to adapt and validate the ESCoT in an Italian population of healthy adults. A translation-back-translation procedure was followed to create and refine the Italian version. Then, 94 healthy adults (47 females, mean age  35 ± 15.9) completed the ESCoT, a battery of conventional social cognition tests (Yoni; Reading the Mind in the Eyes Strange Stories, and Social Norm Questionnaire, SNQ) and measures of intelligence and executive functions. Reliability, convergent validity, and predictors of performance on the ESCoT were examined. Results demonstrated good reliability of the ESCoT and an association between the ESCoT scores and some traditional social cognition tests (Yoni cognitive subscale, SNQ). Hierarchical regression results showed that the ESCoT total score was associated with age. Also, the ESCoT subscore (intrapersonal social norm understanding) was associated with education. These findings support the ESCoT as a valid tool testing social norm understanding, a reliable measure of social cognition for an adult Italian population, and provides further evidence that the ESCoT is sensitive to age- and education-related changes in social cognition, and it is a task not affected by general cognitive functioning.

## Introduction

Social cognition is the processing of relevant stimuli to comprehend individuals and their social interactions (Happé et al., 2017) and mirrors a complex structure of related and interdependent abilities (Adolphs, 2009; Gweon et al., 2012; Henry et al., 2013). These abilities embrace crucial milestones for the life-span development of social functioning (Happé and Frith, 2014), comprising both basic abilities, such as social perception and joint attention, and advanced abilities, such as theory of mind (ToM; Baglio and Marchetti, 2016) and social norm understanding (Bicchieri, 2006; Henry et al., 2016; Legros and Cislaghi, 2020). Impairments in social cognition are often prominent clinical symptoms following brain damage (e.g., traumatic brain injury or stroke) but can also be a central characteristic of the early stages of some neurological conditions, such as behavioral-variant frontotemporal dementia (Kennedy and Adolphs, 2012), and a cardinal feature of autism spectrum disorder (Velikonja et al., 2019), as well as psychiatric disorders, such as schizophrenia (Green et al., 2015; d'Arma et al., 2021) and depression (Weightman et al., 2014).

The most widely studied social cognitive skill is ToM, which is the ability to understand and act on the mental states of others (Baglio and Marchetti, 2016). ToM can be subdivided into two sub-components: affective ToM, the understanding of affective mental states and feelings, and cognitive ToM, the comprehension of cognitive mental states, such as intentions, beliefs, and thoughts (Shamay-Tsoory and Aharon-Peretz, 2007; Henry et al., 2016). Affective ToM is typically assessed using images, cartoons, videos, or stories portraying complex affective states (e.g., Reading the Mind in the Eyes Test, RME; Baron-Cohen et al., 2001a,b). Cognitive ToM tends to be assessed using false-belief tasks where there is a disparity between a protagonist’s understanding of a situation and the participant’s knowledge of reality (e.g., False-Belief task; Gregory et al., 2002). There are also a number of ToM tests purported to assess both cognitive and affective ToM within the same test (e.g., Strange Stories test; Happé et al., 1998; Faux Pas test; Stone et al., 1998). However, these tests were devised before ToM was considered a multidimensional process, and as such, there is no clear distinction between cognitive and affective ToM (Henry et al., 2013).

Another aspect of social cognition that is less commonly assessed clinically is social norm understanding. Social norm understanding refers to the comprehension of shared rules about behaving in a socially acceptable manner (Bicchieri, 2006; Legros and Cislaghi, 2020). Social rules create expectations regarding others’ behavior in specific contexts and support the interpretation of social situations (Carugati and Michel, 1993; Massaro et al., 2014). Therefore, violating a social rule can be detrimental to existing relationships or opportunities to form social relationships. Tests of social norm understanding include the Social Norms Questionnaire (SNQ; Kramer et al., 2014), where participants are asked to indicate whether or not behaviors are socially acceptable in the presence of a stranger or acquaintance (e.g., tell a stranger you do not like their hairstyle).

Social cognition has been formally included in the fifth Diagnostic and Statistical Manual of Mental Disorders (American Psychiatric Association (APA), 2013) as a core cognitive domain that can be affected by a clinical disorder, and social cognition tools should be included in neuropsychological evaluation to highlight any difficulties patients may be experiencing in their social skills. Deficits in social abilities can be more debilitating than traditionally assessed cognitive deficits (Henry et al., 2016). They can have severe psychosocial consequences, such as negatively affecting an individual’s ability to work toward rehabilitation goals, to return to or remain in work, or maintain meaningful social relationships (Ownsworth and McKenna, 2004). Yet, assessing different aspects of social cognition in the clinic can be challenging as social cognition assessments can be lengthy and may focus on one aspect of social cognition. Different social cognition tests vary in their stimuli type and difficulty level (i.e., Happé, 1994; McDonald et al., 2004; Shamay-Tsoory et al., 2007). This can make assessing social cognition difficult for clinicians with limited time who wish to assess an overall understanding of an individual’s social cognitive ability.

Some criticisms of existing social cognition tests are the lack of ecological validity, which limits the ability to closely reflect how we use our social cognitive skills in everyday interactions (Mathersul et al., 2013). Common ToM tests, such as the Faux Pas and Strange Stories, evaluate mentalizing based on short verbal narratives. Although they both provide plenty of contexts for mentalizing reasoning, the fact that social interactions are embedded in verbal stories renders them overly simplified and unimodal (Achim et al., 2013). On the other hand, dynamic visual information portraying a social interaction is more ecologically valid and information-rich than verbal narratives (Henry et al., 2013). For this purpose, new tools have been developed to ensure ecological validity by portraying everyday interactions in a realistic way, such as the Movie for the Assessment of Social Cognition (MASC; Dziobek et al., 2006), the Awareness of Social Inference Test (TASIT; McDonald et al., 2004), the Empathic Accuracy Task (McKenzie et al., 2022), and the Adult Theory of Mind (Brewer et al., 2017). The potential of these tools consists of embedding social situations in a rich real-life scenario, ensuring the multimodal perception related to everyday living. However, although they provide a naturalistic assessment of ToM through video clips representing social situations, they usually have a lengthy administration time (30–60 min). Also, their use is hardly suitable in different cultures due to the presence of verbal dialogs, culture-dependent gestures, and prosody. For instance, the MASC has been dubbed in languages other than German, such as Italian (Fossati et al., 2018), and some contextual information relating to the interactions may have been lost in translation. Recently, the Virtual Assessment of Mentalizing Ability (VAMA; Canty et al., 2017) has also been implemented for the ToM assessment in a first-person virtual scenario, aiming to assess mentalizing reasoning when the subject is immersed in the context. However, it requires participants to have good familiarity with technology. Another concern is that most of the social cognition tools using multimedia content for ecological purposes use forced-choice answers. For instance, many social cognition tools, such as the MASC (Dziobek et al., 2006), and the TASIT (McDonald et al., 2004) have adopted closed-ended questions, for quick and standardized test scoring, but making it difficult to assess individual mental reasoning strategies. In fact, the same level of social cognition ability in terms of equivalent scores on a test in two individuals may reflect different functional or maladaptive strategies for mental reasoning. Instead, an open-ended answer deepens the individual’s model of thought, and thus more finely captures individual differences in mental state understanding. Finally, another relevant issue is that the majority of social cognition tools are affected by cognitive function and demographic characteristics. In fact, evidence has reported that many social cognition tests are influenced by the level of cognitive functions, such as intelligence, and executive functions (e.g., Charlton et al., 2009; Ibanez et al., 2013; Bottiroli et al., 2016), which results in them assessing both social and non-social skills. Moreover, the majority of social cognition tests are affected by demographic variables, such as sex, age, and educational years (Bottiroli et al., 2016; Rosi et al., 2016; Chiasson et al., 2017; Isernia et al., 2020), that rarely are considered in statistical analyses or adjusted according to normative data.

The Edinburgh Social Cognition Test (ESCoT; Baksh et al., 2018) is a new ecological tool based on real-life scenarios presented through dynamic cartoons, which assesses cognitive and affective ToM and social norm understanding. In particular, two aspects of social norm understanding are considered: interpersonal understanding, the ability to comprehend whether a person is behaving following shared social norms in social interaction, and intrapersonal understanding, the ability to understand how you, yourself, would behave in a social interaction based on the specific context and social rules. The test has normative data and cutoffs from a United Kingdom population (Baksh et al., 2018). ESCoT performance has also not been found to be influenced by verbal comprehension, perceptual reasoning, or executive abilities in healthy populations (Baksh et al., 2018, 2020), distinguishing itself against other social cognition tools, where performance is associated with intelligence (Charlton et al., 2009) as well as executive functions (Aboulafia-Brakha et al., 2011). The ESCoT has good validity for diagnosing autism spectrum disorder (Baksh et al., 2021) and excellent sensitivity to social cognition impairments in patients with acquired brain injuries (Poveda et al., 2021). Moreover, the ESCoT offers a simultaneous assessment of both ToM and social norm understanding within the same scenarios, mirroring the complexity of everyday life interactions.

The current work firstly aimed to adapt the ESCoT for the Italian population to provide an ecologically integrated social cognition tool in an Italian context (Baksh et al., 2018, 2020, 2021). We also investigated possible predictors of ESCoT performance, including demographic characteristics, IQ, and executive functions, to detect possible confounding variables.

## Materials and methods

### Participants

Healthy participants were recruited from the IRCCS (Istituto di Ricovero e Cura a Carattere Scientifico) Don Carlo Gnocchi Foundation (Milan) clinic. They were clinicians, researchers, and administrative staff. In addition, participants were recruited from the University of Milan. All participants were volunteers and did not receive compensation for taking part in the research. In total, 94 participants (47 females; age: mean = 35.00 ± 15.90, median = 16.0, range 19–70; years of education: mean = 15.00 ± 2.78, median = 13.0, range 8–23).

The inclusion criteria were: (i) age ≥ 18 years; (ii) an absence of a history of neurological and psychiatric conditions, as reported during a clinical evaluation; (iii) an absence of concurrent oncological and relevant organic conditions; (iv) an absence of cognitive impairment, as assessed by the Montreal Cognitive Assessment (MoCA, Conti et al., 2015); (v) an absence of auditory and visual disability that could affect performance during the assessment; and (vi) an absence of pharmacological treatments affecting cognitive functions.

The study was approved by the Università Cattolica del Sacro Cuore Ethical Committee. All participants gave written informed consent.

### Materials

Participants took part in a single individual session lasting about 2 h. Data collection started in December 2019 and ended in January 2021. Given the occurrence of the COVID-19 pandemic during the research, some of the participants (67%) who were enrolled before the pandemic (December 2019–March 2020) were evaluated in the laboratory by a researcher neuropsychologist, while the rest of the group enrolled during the pandemic in Italy (after March 2020) were tested at home remotely, by a telepresence system, by the same researcher. The same version of the tests was used for people evaluated in the laboratory and at home (more details in Supplementary material S1). The two subsamples’ performances are reported separately in Supplementary material S1.

Participants were assessed using the following measures to evaluate their social cognition abilities and cognitive functions.

#### Social cognition tests

**Figure 1:**
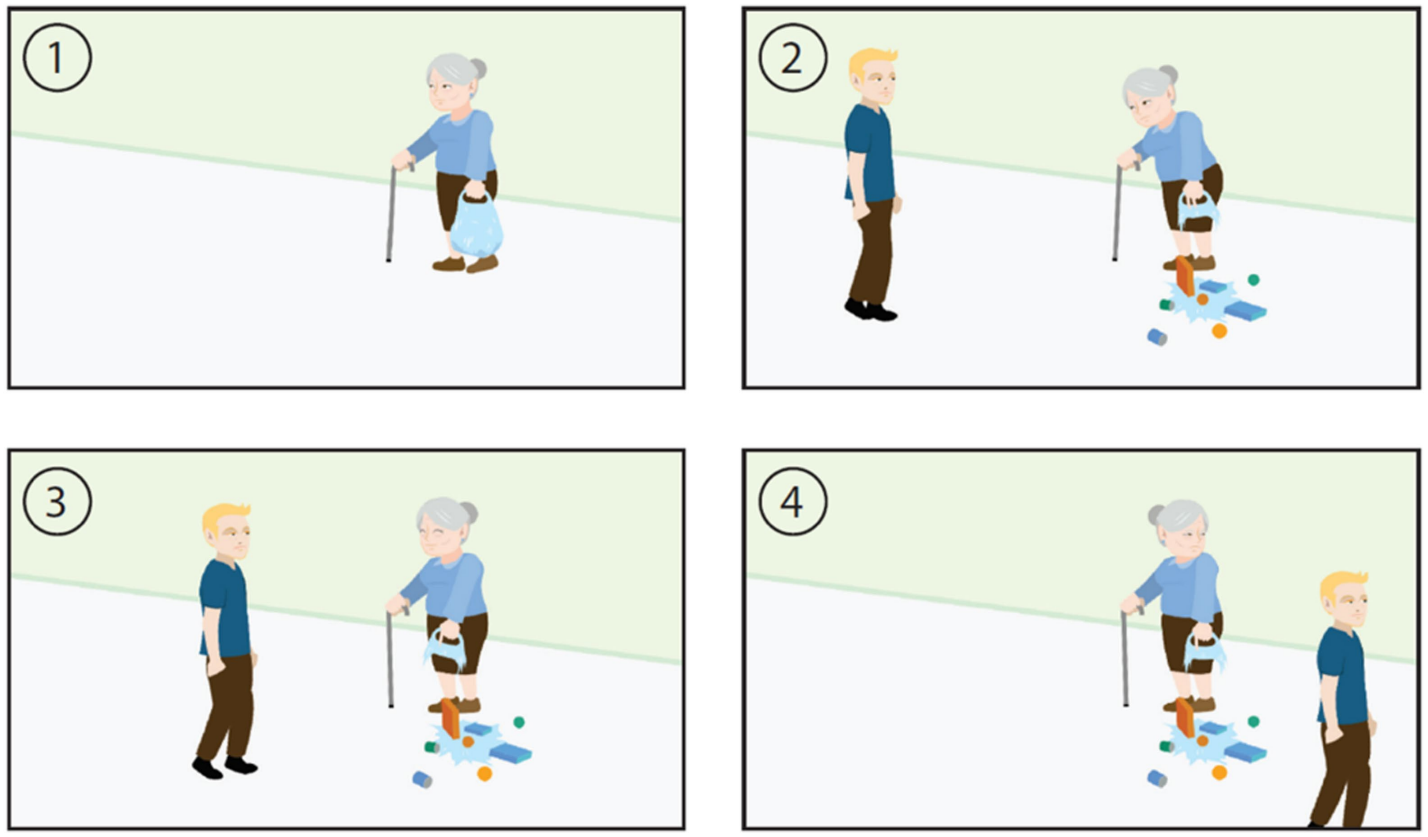
An example interaction of ESCoT.

*The Italian version of the Edinburgh Social Cognition Test* (I_ESCoT). The original English version of the ESCoT (Baksh et al., 2018) measures social cognition by evaluating affective and cognitive ToM and interpersonal and intrapersonal social norm understanding. The test consists of 11 cartoon-style silent animations lasting about 30 s depicting daily life interactions (Figure 1). The animations (including one practice item and 10 test items) show interactions that comply with or violate social norms. The animation is presented in the middle of a computer screen, and at the end of each animation, a static storyboard depicting a summarized version of the interaction is presented. The storyboard remains on the screen for the duration of the trial. Participants are asked to describe what has occurred in the interaction, and then they are asked one question to assess each of the four subtests of social cognition. The I_ESCoT was based on the original English version of the ESCoT (Baksh et al., 2018). The ESCoT, including the manual for administration and scoring, and the answer sheet, was translated into Italian using a translation-back-translation design (Del Greco et al., 1987). A step-by-step procedure was followed: (i) permission to use the ESCoT by its developers was obtained; (ii) a native Italian researcher (resident in Italy), fluent in the English language, performed the forward translation of the original English version of the ESCoT into a provisional Italian version; (iii) a second native Italian researcher (resident in Italy), equally fluent in the English language, rated the Italian translation in terms of clarity, common language usage, and conceptual equivalence; (iv) a native English speaker with fluency in the Italian language carried out the backward translation of the provisional Italian version of the ESCoT into a new English version; (v) the equivalence between the backward translation and the original English version was independently checked by the forward and backward translators; (vi) harmonization meetings involving the forward and backward translators took place; and (vii) issues relating to item translations and conceptual issues which were not solved during the previous steps were managed in a iterative way through new translation-retranslation cycles.

The I_ESCoT was administered in the same way as the English ESCoT. Participants were told they were going to watch animations that told a story and asked questions about what they saw. After participants watched each animation, they were asked five open-ended questions: (1) *“Can you tell me what’s happening in this story, starting with the first picture and finishing with the last picture?”* (animation comprehension), (2) *“What is the character thinking?”* (cognitive ToM); (3) *“How does the character feel at the end of the animation?”* (affective ToM), (4) *“Did the character in the animation behave as other people should behave?”* (interpersonal understanding); and (5) *“Would you have acted the same as the character in the animation?”* (intrapersonal understanding). To allow participants to give their optimal interpretation of each interaction and capture the quality of their response, they were prompted if they gave a limited response or their response lacked important information from the interaction. They were prompted with the question, “Can you tell me more about what you mean by that?” or “Can you explain that in a little bit more detail?.” Each participant was prompted only once for each question. Each response score ranged from 3 (maximum) to 0 (minimum) points. A score of 3 referred to a response that explicitly extracted and integrated the relevant social information and the context related to the interaction depicted in the video. An I_ESCoT score was computed considering cognitive ToM (ToM_C_), affective ToM (ToM_A_), interpersonal social norm understanding (SNU_INTER_), and intrapersonal social norm understanding (SNU_INTRA_). The total score for each interaction ranged from 0 to 12 points, and the total score ranged from 0 to 120. In addition, four subscores were calculated by summing ToM_C_ (0–30), ToM_A_ (0–30), SNU_INTER_(0–30), and SNU_INTRA_ (0–30).

*The Autism-Spectrum Quotient* (AQ; Baron-Cohen et al., 2001a,b). The AQ, adapted to the Italian language (Ruta et al., 2012), is a 50-item self-report questionnaire measuring the presence of autistic traits. Participants were invited to report their agreement with statements from four options (“definitely agree,” “slightly agree,” “slightly disagree,” and “definitely disagree”). Each item was scored 0–1, with a total score ranging from 0 to 50, with higher scores suggesting the presence of more autistic traits.

*The Empathy Quotient* (EQ; Baron-Cohen and Wheelwright, 2004). The EQ, validated in its Italian version (Preti et al., 2011), is a self-administered questionnaire measuring empathy. Participants were invited to report their adherence to empathic behaviors from four options (“strongly agree,” “slightly agree,” “slightly disagree,” and “strongly disagree”). Each item was scored 0–2, with a total score ranging from 0 to 80 and higher scores suggesting a higher level of empathy.

*The Reading the Mind in the Eyes* (RME; Baron-Cohen et al., 2001a,b). The RME measures affective ToM based on the ability to judge mental states from the eyes. The RME test comprises 36 items, black-and-white photographs of the eye region of males and females displaying emotions. The Italian version was used (Maddaluno et al., 2022). The participant was asked to choose the adjective that best fitted the photograph among four alternatives. Each item was scored 0–1, with a total score that ranged from 0 to 36. A gender recognition task was also performed with the same RME items as a control task.

*The Yoni task* (Shamay-Tsoory et al., 2007). The Yoni task is a computerized measure of ToM. It assesses first- and second-order ToM and cognitive and affective ToM. The Italian version of the test consists of 98 trials (Rossetto et al., 2018; Isernia et al., 2022). Each trial showed a face (Yoni) in the center of the computer screen with four objects/faces in the corners of the computer screen. The participant was asked to choose the object/face that Yoni was referring to *via* their eye gaze. Eighty-four items required either a first- or second-order, cognitive or affective inference, while the remaining 14 items required a first- or second-order physical inference (control conditions). Each item was scored 0–1, with a total score (YONI_TOT_) ranging from 0 to 98. Four subtotals were also obtained for the first-order items (YONI_1_: range 0–24), the second-order items (YONI_2_: range 0–60), the cognitive items (YONI_A_: range 0–36), and the affective items (YONI_C_: range 0–48).

*Strange Stories* (Happé, 1994). The strange stories, the Italian version, is an advanced test of affective and cognitive ToM based on the comprehension of stories reporting complex social situations. The Italian version was utilized (Mazzola and Camaioni, 2002; Liverta Sempio et al., 2005). Eight stories from the full version designed by Happé (1994) were used to assess the ability to understand the thoughts and feelings driving behaviors. The social situations presented in the stories comprise: forget, conflicting emotions, sarcasm, metaphors, figures of speech, pretend, white lies, and jokes. The participant listened to a story and then explained the thoughts, intentions, and emotions driving the characters’ behavior. Each answer was rated 0–2: zero was given for an incorrect understanding of the facts in the story or an inappropriate reason for the characters’ behavior; a score of 1 indicated an explanation of the behavior but only in terms of physical and not mental facts; while 2 points were awarded for a response which included the appropriate mental state related to the character’s behavior. The total score ranges from 0 to 16, with a higher score referring to higher ToM abilities.

*Social Norm Questionnaire* (SNQ, Kramer et al., 2014; Panchal et al., 2016). The SNQ is a test measuring the ability to comprehend the implicit social standards in a participant’s mainstream society. The participant judged 22 behaviors as socially appropriate or inappropriate. Two scores were derived from the test: the Break score (SNQ_BREAK_), which consists of the number of errors due to socially inappropriate behaviors judged as acceptable, while the Overadhere (SNQ_OVER_) score was the number of errors due to socially acceptable behaviors being judged as not acceptable. A total score is also computed (range 0–22) by summing the number of correct responses.

#### Neuropsychological tests

*Stroop Test* (Stroop, 1935). The Stroop Test was administered to measure executive functioning, including selective attention, cognitive flexibility, and cognitive inhibition (Strauss et al., 2006; Van der Elst et al., 2006). It consists of three tasks; (i) the word task, (ii) the color-naming task, and (iii) the color-word task. In the word task, participants were invited to read aloud a list of names of colors written in black ink as quickly as possible. In the color-naming task, participants had to name the colors of circles as fast as possible. Finally, in the color-word task, the interference condition, participants were required to name aloud the color of the ink that color words were printed in (e.g., “RED” printed in blue ink) as quickly as possible. The number of errors and the performance time was recorded for each task. Total time and accuracy scores were computed by subtracting the mean of time/number of errors in the word and color-naming tasks from the time/number of errors from the color-word task. For the present study, we adjusted the scores according to Caffarra et al. (2002) and considered only Stroop Time for correlation analyses due to the low occurrence of errors in the healthy adult population.

*Digit Span Backward and Forward* (Monaco et al., 2013). The Digit Span Backward and Forward tests assess working and short-term memory, respectively. The participant listened to a list of numbers and recalled them in the same order (digit *forward*) or the reverse order (digit *backward*) they were presented. The maximum digit sequence length correctly recalled was recorded. In the present study, raw scores were adjusted for age and education according to Monaco et al. (2013).

*Wechsler Adult Intelligence Scale-IV* (WAIS-IV; Wechsler, 2008). Six subtests of the WAIS-IV were administered to assess perceptual reasoning and verbal comprehension: Matrix Reasoning, Block Design, Visual Puzzles, Information, Vocabulary, and Similarities. Following the instructions of Wechsler (2008), raw scores for the separate subtests were adjusted, and the Perceptual Reasoning Index (PRI) and Verbal Comprehension Index (VCI) were computed. People evaluated during the Severe Acute Respiratory Syndrome Coronavirus-2 (SARS-COV2) pandemic remotely did not perform the Block Design. For this group, the PRI was derived following Wechsler’s instructions (see Supplementary material S1, for more details).

### Statistical analysis

Jamovi 1.2.27 was used for the statistical analysis.

Summary statistics, including means, frequencies, and standard deviations, were calculated to explore the demographic characteristics of the sample.

*Selection of the I_ESCoT items:* Single item accuracy means and standard deviations and item-rest correlations were computed. Cronbach’s alpha and McDonald’s omega were computed when items were removed, or ambiguous items were excluded.

*Evaluation of the I_ESCoT reliability:* The internal consistency of the scale (Cronbach’s alpha and McDonald’s omega) was computed. In addition, the inter-rater reliability coefficient (2 raters) was computed on the entire sample.

*Evaluation of the I_ESCoT validity:* The ESCoT association with conventional social cognition measures (Yoni, RME, Strange Stories, SNQ, EQ, and AQ) were assessed by running partial correlations (covariate: the period of study participation - before or during the pandemic). In addition, partial correlations between other social cognition tests were explored.

*Potential predictors of I_ESCoT performance:* The effects of cognitive functions, intelligence, and demographical variables were investigated by performing multiple regression models. Two blocks of variables were hierarchically inserted into the model using the enter method (first block: age, gender, years of education; second block: digit forward, digit backward, Stroop, VCI, and PRI).

The *value of p* threshold was set according to the False Discovery Rate (*p*_FDR_) multiple comparison correction (Benjamini-Hochberg).

Given the COVID-19 pandemic occurrence during the research, the period of study participation (before or during the pandemic) was considered in all the analyses as a covariate.

The sample size was based on a previous study validating the original version of ESCoT in the United Kindom population (Baksh et al., 2018). In this study, a sample of 91 healthy subjects was sufficient to prove the convergent validity (correlation of ESCoT with RME, *p* < 0.01), inter-rater reliability (ICC = 0.90), and predictors of ESCoT (age, *p* < 0.001).

## Results

### Item pool and reliability of the I_ESCoT

After considering the means and standard deviations for item accuracy, item-rest correlations (r ≥ 0.3), and Cronbach’s alpha/McDonald’s omega (scale Cronbach’s alpha ≥ Cronbach’s alpha when item dropped/scale McDonald’s omega ≥ McDonald’s omega when item dropped), items 1 (Helping the elderly), 3 (Being considerate on the bus), 4 (Cleaning up after your pet), 6 (Smoking in a prohibited area), 7 (Talking in the cinema), 8 (Serving a customer), 9 (Skipping a bus queue), and 10 (Assisting a stranger) were included in the I_ESCoT. Items 2 (Disobeying parking regulations) and 5 (Assisting a neighbor) were excluded from the item pool, both showing a poor correlation with the other I_ESCoT items (*r* < 0.30) and an increment in Cronbach’s alpha/McDonald’s omega when dropped. The 8-item Italian version of the I_ESCoT had a Cronbach’s alpha of 0.70 and a McDonald’s omega of 0.70, with an item mean accuracy of 8.94 ± 0.80. The inter-rater reliability was high (I_ESCoT total: ICC = 0.94; ToM_C_: ICC = 0.92; ToM_A_: ICC = 0.94; SNU_INTER_: ICC = 0.90; SNU_INTRA_: ICC = 0.98).

### Performance on social cognition tools and cognitive assessment

Participants’ performance on social cognition tests and cognitive function measures is reported in Table 1. I_ESCoT performance in the participants group evaluated in the laboratory and at home is reported in Supplementary material S1.

**Table 1:** Participants’ performance on social cognition and neuropsychological tests.

Domain	Scale	Scale range	M, SD	Median	Skewness	Kurtosis	Kolmogorov–Smirnov *p*-value
Social cognition	ESCoT total	0–96	71.50, 6.44	72.00	−0.29	−0.61	0.69
	ESCoT ToM_C_	0–24	15.90, 1.73	16.00	0.30	−0.15	0.05
	ESCoT ToM_A_	0–24	18.30, 4.09	19.00	−0.93	0.05	0.36
	ESCoT SNU_INTER_	0–24	15.90, 1.99	16.00	0.25	−0.65	0.45
	ESCoT SNU_INTRA_	0–24	21.40, 2.10	21.00	−0.44	−0.36	0.14
	RME	0–36	26.70, 3.53	27.00	−0.50	0.70	0.62
	YONI_TOT_	0–98	71.90, 12.6	76.50	−1.72	2.80	0.00
	YONI_C_	0–36	30.30, 6.26	33.00	−1.72	2.95	0.00
	YONI_A_	0–48	41.60, 6.82	44.00	−1.55	2.13	0.00
	YONI_1_	0–24	22.00, 4.17	24.00	−2.79	7.72	0.00
	YONI_2_	0–60	50.00, 9.41	53.00	−1.24	0.70	0.00
	SS	0–16	12.90, 1.64	13.00	−0.56	0.86	0.13
	AQ	0–50	16.10, 6.30	15.00	0.21	−0.28	0.48
	EQ	0–80	47.00, 8.96	46.00	0.25	0.40	0.51
	SNQ total	0–22	18.40, 1.82	19.00	−0.43	−0.41	0.04
	SNQ_BREAK_	0–22	1.76, 1.54	1.00	1.33	2.05	0.00
	SNQ_OVER_	0–22	1.84, 1.45	2.00	0.93	1.02	0.03
Cognitive functions	Digit forward	0–9	5.89, 1.22	5.44	0.58	0.11	0.00
	Digit backward	0–9	4.68, 1.17	4.42	0.51	0.18	0.15
	Stroop time(s)	–	21.60, 8.38	22.30	−0.41	−0.24	0.98
	Stroop errors	–	0.30, 1.03	0.00	3.54	11.70	0.00
	Verbal comprehension index	47–153	99.00, 11.50	98.00	−0.00	−0.69	0.28
	Perceptual reasoning index	47–156	105.00, 16.20	104.00	0.02	−0.29	0.77

AQ, Autism quotient; EQ, Empathy quotient; ESCoT, Edinburgh Social Cognition Test; M, Mean; RME, Reading the mind in the eyes test; SD, Standard deviation; SNQ, Social norm questionnaire; SS, Strange stories.

### Correlations between the I_ESCoT subscores

Statistically significant associations were found between all I_ESCoT subscores and the total score, as well as ToM_C_ and ToM_A_ (see Supplementary Table S2.2).

### Convergent validity and predictors of I_ESCoT performance

Significant correlations were found between I_ESCoT scores and YONI_C_ (I_ESCoT ToM_C_: *rho* = 0.328, *p*_FDR_ = 0.012), SNQ_TOT_ (I_ESCoT SNU_INTRA_: *rho* = 0.339, *p*_FDR_ = 0.001), and SNQ_BREAK_ (ESCoT SNU_INTRA_: *rho* = − 0.278, *p*_FDR_ = 0.042). No significant correlation between I_ESCoT and RME (*r* = 0.112), Strange Stories (*r* = 0.150), AQ (*r* = −0.005), and EQ (*r* = 0.077) was observed (see Table 2). Supplementary material S2 reports associations among the other social cognition tests.

**Table 2:** Partial correlation analysis (covariate: the period of study participation—before or during the pandemic) between ESCoT subscores and social cognition tests.

	ESCoT_TOT_	ToM_C_	ToM_A_	SNU_INTER_	SNU_INTRA_
RME (r, *p_FDR_*)	0.112, 0.430	0.079, 0.321	−0.029, 0.934	0.210, 0.264	0.068, 0.775
SS (r, *p_FDR_*)	0.150, 0.302	−0.010, 0.985	0.169, 0.630	0.130, 0.843	0.009, 0.934
YONI (rho, *p_FDR_*)	0.166, 0.302	0.259, 0.056	−0.020, 0.934	0.047, 0.843	0.142, 0.392
YONI_C_ (rho, *p_FDR_*)	0.216, 0.152	0.328, 0.012	−0.012, 0.934	0.065, 0.843	0.189, 0.280
YONI_A_ (rho, *p_FDR_*)	0.124, 0.404	0.182, 0.140	0.009, 0.934	0.014, 0.894	0.097, 0.605
YONI_1_ (rho, *p_FDR_*)	0.015, 0.964	0.190, 0.136	−0.108, 0.915	−0.035, 0.843	0.027, 0.934
YONI_2_ (rho, *p_FDR_*)	0.160, 0.302	0.253, 0.056	−0.017, 0.934	0.044, 0.843	0.135, 0.392
SNQ total (rho, *p_FDR_*)	0.229, 0.152	0.195, 0.136	0.013, 0.934	0.083, 0.843	0.339, 0.001
SNQ_BREAK_ (rho, *p_FDR_*)	−0.224, 0.152	−0.198, 0.136	−0.046, 0.934	−0.030, 0.843	−0.278, 0.042
SNQ_OVER_ (rho, *p_FDR_*)	−0.077, 0.558	−0.033, 0.823	0.027, 0.934	−0.076, 0.843	−0.148, 0.392
AQ (r, *p_FDR_*)	−0.005, 0.964	−0.043, 0.816	0.126, 0.915	−0.234, 0.264	0.021, 0.934
EQ (r, *p_FDR_*)	−0.077, 0.558	0.111, 0.387	−0.218, 0.432	0.070, 0.843	0.009, 0.934

r Pearson correlation coefficient was reported; rho Spearman correlation coefficient was reported; *p*-value was adjusted for FDR correction. AQ, Autism Quotient; EQ, Empathy Quotient; RME, Reading the Mind in the Eyes Test; ESCoT, Edinburgh Social COgnition Test; ToM_C_, Edinburgh Social COgnition Test cognitive theory of mind subscore; ToM_A_, Edinburgh Social COgnition Test affective theory of mind subscore; SNQ, Social Norm Questionnaire total score; SNQ_BREAK_, Social Norm Questionnaire break subscore; SNQ_OVER_, Social Norm Questionnaire overadherence subscore; SNU_INTER_, Edinburgh Social COgnition Test interpersonal social norm understanding subscore; SNU_INTRA_, Edinburgh Social COgnition Test intrapersonal social norm understanding subscore; SS, Strange Stories; Yoni_1_, Yoni first-order ToM subscore; Yoni_2_,Yoni second-order ToM subscore; Yoni_A_, Yoni affective ToM subscore; Yoni_C_, Yoni cognitive ToM subscore.

Multiple regression models (Model 1: age, years of education, gender; Model 2: age, years of education, gender, digit forward, digit backward, Stroop time, VCI, and PRI) revealed age as a significant predictor of performance on the I_ESCoT total score (Table 3) where the higher the age, the poorer the performance. Instead, for I_ESCoT ToM_C_, even if gender was reported as the significant predictor, where women performed better than men, the overall model did not significantly differ from a null model (Table 4). Regarding the I_ESCoT SNU_INTRA_ score, both age and education had a predictive role on performance (Table 7), whereas younger age and higher education were associated with better performance. No significant predictors were highlighted in I_ESCoT SNU_INTER_ (Table 6)_._ For the I_ESCoT total score, ToM_A,_ and SNU_INTRA,_ the administration period (before/during the pandemic) was a significant predictor of the performance (see Tables 5, 7). Multiple regression models identifying predictors on other social cognition tests are reported in Supplementary Table S3–S8.

**Table 3:** Multiple regression results for predictors of ESCoT total score.

	Predictors on ESCoT	*β*	S.E.	*t*	*p*-value	*F*	*Omnibus* *p*-value
Model 1 R^2^ = 0.20	Age	−0.04	0.01	−2.38	0.020	5.51	<0.001
	Years of education	0.00	0.00	1.76	0.082		
	Gender (males = 1, females = 2)	−0.01	0.01	−0.40	0.694		
	Participation before/during pandemic (covariate)	0.07	0.02	4.52	<0.001		
	Intercept	0.65	0.04	16.90	<0.001		
Model 2 R^2^ = 0.25	Age	−0.05	0.02	−2.40	0.019		
	Years of education	0.01	0.00	1.79	0.078		
	Gender (males = 1, females = 2)	−0.00	0.01	−0.31	0.760	3.16	0.003
	Digit forward	−0.01	0.01	−0.88	0.381		
	Digit backward	0.01	0.01	1.38	0.172		
	Stroop time	−0.00	0.00	−1.29	0.200		
	VCI	−0.00	0.00	−0.82	0.417		
	PRI	−0.00	0.00	0.73	0.466		
	Participation before/during pandemic (covariate)	0.06	0.02	2.96	0.004		
	Intercept	0.70	0.08	8.56	<0.001		

Period of research participation (before or during the pandemic period) was entered in the model as a covariate. PRI, perceptual reasoning index; VCI, verbal comprehension reasoning.

**Table 4:** Multiple regression results for predictors of ESCoT ToM_C_ total score.

	Predictors on ESCoT ToM_C_	*β*	S.E.	*t*	*p*-value	*F*	*Omnibus* *p*-value
Model 1 R^2^ = 0.06	Age	−0.02	0.02	−0.86	0.393	1.32	0.267
	Years of education	0.00	0.00	0.51	0.613		
	Gender (males = 1, females = 2)	0.03	0.01	2.16	0.033		
	Participation before/during pandemic (covariate)	0.01	0.02	0.33	0.740		
	Intercept	0.64	0.04	14.23	<0.001		
Model 2 R^2^ = 0.10	Age	−0.02	0.03	−0.92	0.362	0.97	0.468
	Years of education	−0.00	0.00	−0.17	0.862		
	Gender (males = 1, females = 2)	0.04	0.02	2.45	0.016		
	Digit forward	0.00	0.01	0.28	0.781		
	Digit backward	0.01	0.01	0.82	0.412		
	Stroop time	−0.00	0.00	−0.24	0.812		
	VCI	0.00	0.00	0.24	0.813		
	PRI	0.00	0.00	1.05	0.295		
	Participation before/during pandemic (covariate)	−0.00	0.02	−0.04	0.964		
	Intercept	0.56	0.10	5.49	<0.001		

Period of research participation (before or during the pandemic period) was entered in the model as a covariate. PRI, perceptual reasoning index; VCI, verbal comprehension reasoning.

**Table 5:** Multiple regression results for predictors of ESCoT ToM_A_ total score.

	Predictors on ESCoT ToM_A_	*β*	S.E.	*t*	*p*-value	*F*	*Omnibus* *p*-value
Model 1 R^2^ = 0.21	Age	−0.02	0.04	−0.71	0.480	5.71	<0.001
	Years of education	0.00	0.01	0.53	0.599		
	Gender (males = 1, females = 2)	−0.05	0.03	−1.41	0.161		
	Participation before/during pandemic (covariate)	0.17	0.04	4.23	<0.001		
	Intercept	0.66	0.10	6.83	<0.001		
Model 2 R^2^ = 0.28	Age	−0.08	0.05	−1.52	0.133	3.55	<0.001
	Years of education	0.01	0.01	1.29	0.202		
	Gender (males = 1, females = 2)	−0.04	0.03	−1.16	0.251		
	Digit forward	0.00	0.02	0.10	0.920		
	Digit backward	0.01	0.02	0.69	0.492		
	Stroop time	−0.00	0.00	−1.84	0.069		
	VCI	−0.00	0.00	−1.84	0.069		
	PRI	0.00	0.00	0.73	0.470		
	Participation before/during pandemic (covariate)	0.12	0.05	2.49	0.015		
	Intercept	0.99	0.21	4.64	<0.001		

Period of research participation (before or during the pandemic period) was entered in the model as a covariate. PRI, perceptual reasoning index; VCI, verbal comprehension reasoning.

**Table 6:** Multiple regression results for predictors of ESCoT SNU_INTER_ total score.

	Predictors on ESCoT SNU_INTER_	*β*	S.E.	*t*	*p*-value	*F*	*Omnibus* *p*-value
Model 1 R^2^ = 0.04	Age	−0.03	0.02	−1.26	0.211	0.91	0.464
	Years of education	0.00	0.05	1.32	0.191		
	Gender (males = 1, females = 2)	−0.01	0.02	−0.35	0.730		
	Participation before/during pandemic (covariate)	0.03	0.02	1.39	0.169		
	Intercept	0.62	0.05	11.85	<0.001		
Model 2 R^2^ = 0.14	Age	−0.02	0.03	1.32	0.189	0.84	0.581
	Years of education	0.00	0.00	0.92	0.358		
	Gender (males = 1, females = 2)	−0.01	0.02	−0.63	0.530		
	Digit forward	−0.01	0.01	−1.30	0.198		
	Digit backward	0.01	0.01	1.32	0.190		
	Stroop time	0.00	0.00	0.30	0.764		
	VCI	0.00	0.00	1.01	0.313		
	PRI	−0.00	0.00	−1.03	0.303		
	Participation before/during pandemic (covariate)	0.03	0.03	1.32	0.189		
	Intercept	0.58	0.12	4.91	<0.001		

Period of research participation (before or during the pandemic period) was entered in the model as a covariate. PRI, perceptual reasoning index; VCI, verbal comprehension reasoning.

**Table 7:** Multiple regression results for predictors of ESCoT SNU_INTRA_ total score.

	Predictors on ESCoT SNU_INTRA_	*β*	S.E.	*t*	*p*-value	*F*	*Omnibus* *p*-value
Model 1 R^2^ = 0.21	Age	−0.08	0.02	−3.91	<0.001	5.92	<0.001
	Years of education	0.01	0.00	2.59	0.011		
	Gender (males = 1, females = 2)	−0.00	0.02	−0.06	0.955		
	Participation before/during pandemic (covariate)	0.08	0.02	4.01	<0.001		
	Intercept	0.83	0.05	16.74	<0.001		
Model 2 R^2^ = 0.26	Age	−0.09	0.03	−3.01	0.003	3.16	0.002
	Years of education	0.01	0.00	2.22	0.029		
	Gender (males = 1, females = 2)	−0.01	0.02	−0.29	0.768		
	Digit forward	−0.01	0.01	−1.80	0.076		
	Digit backward	0.01	0.01	0.77	0.442		
	Stroop time	−0.00	0.00	−0.54	0.588		
	VCI	−0.00	0.00	−0.27	0.789		
	PRI	0.00	0.00	1.00	0.322		
	Participation before/during pandemic (covariate)	0.07	0.02	2.99	0.004		
	Intercept	0.87	0.11	7.84	<0.001		

Period of research participation (before or during the pandemic period) was entered in the model as a covariate. PRI, perceptual reasoning index; VCI, verbal comprehension reasoning.

## Discussion

The ESCoT is a novel tool for assessing ToM and social norm understanding using dynamic ecological scenarios for the English-speaking population (Baksh et al., 2018, 2020, 2021; Poveda et al., 2021). In the present work, we translated and adapted the Italian version of the ESCoT (I_ESCoT) to a population of healthy Italian adults.

An item pool of eight scenarios was selected to assure adequate reliability of the task in terms of internal consistency (Cronbach’s alpha ≥ 0.70, McDonald’s omega ≥ 0.70) and inter-rater reliability (ICC ≥ 0.90). In detail, the original items 2 and 5, depicting a woman disobeying parking regulations and a man assisting the neighbor needing help to get her cat from the tree, were dropped. This is because the social norm depicted in these two items may be less evident in Italian than in British culture. Especially disobeying a parking regulation would be mainly associated with a non-social norm, especially in a context where no other people need parking in that area. Also, helping a neighbor to get the pet from the tree is related to a possibly dangerous action for the person, the reason why calling the fireman/police is the usual norm.

Interestingly, while affective and cognitive ToM performance within the I_ESCoT scenarios were linked, we did not find a significant association between ToM and social norm understanding scores. Our results may suggest, in accordance with previous studies, that the cognitive and affective components of ToM overlap (Kalbe et al., 2010; Shamay-Tsoory et al., 2010; Sebastian et al., 2011), and that the understanding of the mental states may not explain an individual’s moral reasoning related to socially respectful behaviors. I_ESCoT may consist of a composite social cognition tool, evaluating two distinct social processes, such as ToM and social norm understanding, both relevant when assessing a social cognitive deficit that rarely occurs in isolation (Henry et al., 2016).

Considering the tool’s validity, we reported significant associations of I_ESCoT with social norm understanding, in accordance with previous findings (e.g., Baksh et al., 2018) and the Yoni cognitive ToM subscore. I_ESCoT social norm understanding subscores were associated with the SNQ, which assesses social norm understanding. These findings support the notion that the Italian version of the ESCoT is a valid measure of social norm understanding. However, a single task assessing social norm understanding has been administered, and further studies should validate I_ESCoT against other tests of social norm understanding to confirm this conclusion. Concerning ToM, performance on the I_ESCoT was associated only with the Yoni cognitive ToM score and not with other conventional tests. These results only partly highlight the convergent validity of I_ESCoT as a test assessing ToM. We did not observe an association between performance on the I_ESCoT and RME or Strange Stories. It has to be mentioned that the social processes assessed by RME are currently under debate, with some authors arguing it is a test of emotion recognition (Oakley et al., 2016). In addition, the lack of an association between the Strange Stories and the I_ESCoT may be ascribed to the different features of the tests’ stimuli (i.e., verbal story-based versus dynamic cartoon-like scenarios). Previous work on the ESCoT did not include Strange Stories (Baksh et al., 2018, 2020, 2021; Poveda et al., 2021), so this is the first study to examine the relationship between performance on the ESCoT and Strange Stories. On the other hand, previous studies demonstrated the difficulty of capturing coherence among ToM measures varying across stimuli features in terms of modality, complexity, affective content (Warnell and Redcay, 2019), and no clear empirical evidence supporting common construct validity among different advanced ToM tasks (Hayward and Homer, 2017). In light of these findings, some work depicts the ToM construct as an interactive process spanning multiple cognitive abilities (Apperly, 2012). In this perspective, the association between different ToM tasks may be related to the specific tool’s non-ToM ability demands (Schaafsma et al., 2015). Story-based mental reasoning and the recognition of mental states from photographs may involve different non-ToM cognitive processes rather than a video-based cartoon-like test such as the ESCoT.

The lack of association between I_ESCoT, AQ, and EQ in the healthy Italian subjects included in the study was also unexpected. However, our sample’s level of autistic traits was very low, which would partly explain the lack of association between these tests and I_ESCoT. Moreover, the other social cognitive tasks did not relate to AQ and EQ. Therefore, future studies mainly focused on the role of autistic phenotype on ESCoT performance, including healthy people with low and high autistic traits, should be carried out.

When examining potential predictors of I_ESCoT total score, we found that age was the only demographic variable influencing performance. The influence of age on I_ESCoT performance is in line with previous work (Baksh et al., 2018, 2020), where higher ESCoT scores were associated with younger age. Considering the subscores of I_ESCoT, age and education were predictors of I_ESCoT SNU_INTRA_. The influence of age on social cognition in the literature is reported (Moran, 2013; Bernstein et al., 2017; Klindt et al., 2017; Fernandes et al., 2021). However, although evidence suggests age negatively affects social cognitive abilities (Bailey et al., 2008; Bailey and Henry, 2008; Duval et al., 2011; Phillips et al., 2011; Cavallini et al., 2013; Bottiroli et al., 2016), education (Li et al., 2013) and the age-related cognitive functions decline might mediate and moderate the performance (Rakoczy et al., 2012).

Our finding that sociodemographic variables were the only predictors of I_ESCoT contrasts with other studies that showed an association between social cognition performance and IQ (e.g., Charlton et al., 2009) or cognitive functions, including executive functions or working memory (e.g., Ibanez et al., 2013). The contrast between these results may be explained in terms of the different characteristics of the social cognition tools adopted, comprising both verbal and silent, static and dynamic stimuli, and closed and open-ended answers. Moreover, earlier work involving healthy individuals did not find associations between ESCoT performance and IQ or executive functions (Baksh et al., 2018, 2020), which is an advantage of the ESCoT over other tests of social cognition. This may be because dynamic cartoon-style social interactions are more ecologically valid and information-rich than verbal narratives, allowing perceivers to use many more cues to make inferences similar to real life (Moran, 2013). However, our sample only included younger and middle-aged people, possibly preventing us from finding associations between I_ESCoT performance and some predictors such as executive functions, which decline in older age (West, 1996; MacPherson et al., 2002; Argiris et al., 2020), especially from 50 to 65 years old (Belghali et al., 2020).

Finally, even if the occurrence of the pandemic emergency in Italy was not the focus of the present study, we observed a significant effect of the test administration period on I_ESCoT performance. In detail, we found that the I_ESCoT affective ToM and the intrapersonal social norm understanding scores were significantly higher during the pandemic emergency (also the lockdown period in Italy). Different research highlighted a significant impact of the pandemic restrictions on social cognition performance (Carbon, 2020; Saunders et al., 2021), mostly regarding reduced social skills. However, these studies mainly focused on personal protective equipment’s effects on emotion recognition capacity, unlike the ESCoT, which focus on ToM reasoning on social situations strictly linked to social norms. It is plausible to assume that a period of reduced social contact due to the proximity-related risk of infection may enhance the acknowledgment of the relevance of social norms. Notably, the ESCoT scenarios are based on social norms that are violated or not and may similarly intensify the comprehension of the affective mental states of individuals involved in that context. In these terms, the eight sources of information framework of mentalizing (8-SIF; Achim et al., 2013) attributed a crucial role of stored information about specific context for ToM performance.

This study is not without its limitations. Our sample does not include the entire age range, and future contributions are needed to validate the Italian version of the ESCoT in older populations. This would allow us to derive age-adjusted scores, as previously provided for the original version of the ESCoT, which presents the opportunity to use the tool in a clinical context. Also, the limited sample size of the present study partially allows us to test the tool’s validity: no construct and divergent validity has been investigated. Future work with a wider sample size must be performed for this purpose. Additionally, our sample mostly consists of people with high levels of education, which is not fully representative of the Italian population. This may limit the generalizability of our results to people with a lower level of education. Finally, the SARS-COV2 pandemic during data collection forced us to rely on unconventional online administration procedures (telepresence platforms) to avoid in-person testing. We considered this variable in our analyses as a covariate and found it to be significantly associated with performance. Examining the influence of the administration modality on ESCoT performance was not a primary aim of this study. Still, it merits further investigation in future studies in larger samples since modality is an important factor when administering neuropsychological tests.

In conclusion, this study presented the Italian adaptation of the ESCoT, a novel valid tool testing social norm understanding and a reliable measure of social cognition, where performance is not influenced by executive function, working memory, or IQ. The ESCoT stands out from other social cognition tests for its multidimensional design to simultaneously measure multiple social cognition processes. The ESCoT may provide researchers and clinicians with an objective measurement of different aspects of social cognition, such as interpersonal, intrapersonal social norm understanding, and cognitive ToM needed to interact with others. This is particularly useful in clinical settings where the results can be used to customize rehabilitation or teach caregivers about the difficulties a patient might be experiencing in processing social information and interacting with others.

## Data availability statement

The raw data supporting the conclusions of this article will be made available by the authors, without undue reservation.

## Ethics statement

The studies involving human participants were reviewed and approved by Università Cattolica del Sacro Cuore Ethical Committee. The patients/participants provided their written informed consent to participate in this study.

## Author contributions

DM, FB, and SI: conceptualization. SI: data curation, formal analysis, and writing—original draft. NB and SI: methodology. DM, FB, and AM: supervision. SM, RB, NB, FB, DM, and AM: review and editing. All authors contributed to the article and approved the submitted version.

## Funding

This work was supported by: 5x1000 funds - 2020, Italian Ministry of Health – Ricerca Corrente; and by Lombardy Region (Announcement POR-FESR 2014–2020—Azione I.1.B.1.3), within the project named Smart&TouchID.

## Conflict of interest

The authors declare that the research was conducted in the absence of any commercial or financial relationships that could be construed as a potential conflict of interest.

## Publisher’s note

All claims expressed in this article are solely those of the authors and do not necessarily represent those of their affiliated organizations, or those of the publisher, the editors and the reviewers. Any product that may be evaluated in this article, or claim that may be made by its manufacturer, is not guaranteed or endorsed by the publisher.
