# Supplementary material for: Italian adaptation of the Edinburgh Social Cognition Test (ESCoT): A new tool for the assessment of theory of mind and social norm understanding
Source: Front Psychol. 2022 Oct 28;13:971187. doi: 10.3389/fpsyg.2022.971187 (PMC9651931; doi:10.3389/fpsyg.2022.971187)
Supplement: Supplementary file 1 [file Data_Sheet_1.docx]

Supplementary Material

Italian adaptation of the Edinburgh Social Cognition Test (ESCoT): a new tool for the assessment of theory of mind and social norm understanding.

**S1. Descriptive statistics of ESCoT evaluations in the laboratory (face-to-face) and at home remotely.**

Data collection started in December 2019 and ended in January 2021. Given the occurrence of the COVID-19 pandemic during the research, part of the participants (67%), who were enrolled before the pandemic (December 2019-March 2020) were evaluated in presence in the laboratory, while the rest of the group, enrolled during the pandemic in Italy (after March 2020) were tested at home remotely, by a telepresence system.

For social cognition tests, the AQ, EQ, and SNQ were self-administered in the laboratory and remotely. For the evaluation at home, these questionnaires were administered by sending participants an online link. Both at home and in the laboratory, while participants filled out these tests, they had the possibility to ask for clarifications from the researcher that was in their presence or telepresence. The Yoni task and the RME were computerized, and both participants were evaluated in the laboratory, and the ones at home used the same link to perform the task through an online platform. The Strange Stories were administered by the researcher in the laboratory and through the telepresence system.

For neuropsychological tests, the same versions of the tests were administered in the laboratory and at home. The administration of the tests remotely was performed by a researcher neuropsychologist that was trained in the Italian Uniform Data Set procedures (Weintraub et al., 2018). The only difference between the participants at home and in the laboratory consisted that people evaluated remotely did not perform the Block Design. For this group, the PRI was derived strictly following Wechsler’s instructions.

|  | **Evaluation in lab** | **Evaluation at home** | **Independent t-test *p-value*** |
| --- | --- | --- | --- |
| **ESCoT tot (M, sd)** | 68.2, 7.22 | 73.1, 5.38 | <0.001 |
| **ToM_C_** | 15.9, 1.83 | 15.9, 1.70 | 0.937 |
| **ToM_A_** | 15.9, 5.08 | 19.5, 2.88 | <0.001 |
| **SNU_INTER_** | 15.6, 2.01 | 16.0, 1.99 | 0.441 |
| **SNU_INTRA_** | 20.8, 1.48 | 21.7, 2.29 | 0.034 |

**Table S1. ESCoT, Edinburgh Social Cognition Test; ToM_C_, ESCoT cognitive ToM; ToM_A_, ESCoT affective ToM; SNU_INTER_, ESCoT inter-personal social norm understanding; SNU_INTRA_, ESCoT intra-personal social norm understanding.**

**S2. Correlations between social cognition tests.**

Partial correlations (covariate: the period of study participation) between other social cognition measures showed significant associations between SS and YONI_AFF_ (rho = 0.235, *p* = 0.024). SNQ revealed an association with YONI_COG_ (rho = 0.224, *p* = 0.031), EQ (r = 0.213, *p* = 0.041), and AQ (r = -0.257, *p* = 0.013). Finally, a significant correlation between SNQ_BREAK_ and EQ (rho = -0.226, *p* = 0.030) was found.

Partial correlations (covariate: the period of study participation) between ESCoT subscores showed a statistically significant association between ToM_C_ and ToM_A._

|  | **SS** | **RME** | **YONI** | **YONI_AFF_** | **YONI_COG_** | **YONI_1_** | **YONI_2_** | **AQ** | **EQ** | **SNQ** | **SNQ_BREAK_** |
| --- | --- | --- | --- | --- | --- | --- | --- | --- | --- | --- | --- |
| **RME** | .019  (.855) |  |  |  |  |  |  |  |  |  |  |
| **YONI** | .174  (.095) | .136  (.193) |  |  |  |  |  |  |  |  |  |
| **YONI_A_** | **.235**  **(.024)** | .170  (.102) | **.967**  **(<.001)** |  |  |  |  |  |  |  |  |
| **YONI_C_** | .091  (.387) | .072  (.494) | **.962**  **(<.001)** | **.715**  **(<.001)** |  |  |  |  |  |  |  |
| **YONI_1_** | .146  (.163) | -.033  (.753) | **.698**  **(<.001)** | **.700**  **(<.001)** | **.620**  **(<.001)** |  |  |  |  |  |  |
| **YONI_2_** | **.192**  **(.066)** | .170  (.103) | **.980**  **(<.001)** | **.905**  **(<.001)** | **.897**  **(<.001)** | .**592**  **(<.001)** |  |  |  |  |  |
| **AQ** | -.067  (.524) | -.132  (.207) | .146  (.163) | .119  (.256) | .154  (.140) | .162  (.122) | .137  (.192) |  |  |  |  |
| **EQ** | -.168  (.108) | -.096  (.362) | .067  (.525) | -.003  (.975) | .125  (.233) | -.088  (.401) | .067  (.525) | **-.431**  **(<.001)** |  |  |  |
| **SNQ** | .154  (.140) | **.202**  **(.052)** | .152  (.145) | .098  (.352) | **.224**  **(.031)** | .021  (.842) | .161  (.122) | **-.241**  **(.020)** | **.229**  **(.027)** |  |  |
| **SNQ_BREAK_** | .060  (.570) | -.083  (.430) | -.074  (.482) | -.025  (.812) | -.125  (.234) | .068  (.516) | -.108  (.304) | **.197**  **(.058)** | **-.226**  **(.030)** | **-.571**  **(<.001)** |  |
| **SNQ_OVER_** | -.181  (.083) | -.122  (.245) | -.091  (.385) | -.059  (.576) | -.148  (.156) | -.006  (.953) | -.087  (.406) | .040  (.702) | -.066  (.528) | **-.590**  **(<.001)** | **-.234**  **(.024)** |

**Table S2.1. Partial correlations between conventional social cognition measures. AQ, Autism Quotient; EQ, Empathy Quotient; RME, Reading the Mind in the Eyes Test; SNQ, Social Norm Questionnaire total score; SNQ_BREAK_, Social Norm Questionnaire break subscore; SNQ_OVER_, Social Norm Questionnaire overadherence subscore; SS, Strange Stories; Yoni_1_, Yoni first-order ToM subscore; Yoni_2_, Yoni second-order ToM subscore; Yoni_A_, Yoni affective ToM subscore; Yoni_C_, Yoni cognitive ToM subscore.**

|  | **ToM_C_** | **ToM_A_** | **SNU_INTER_** | **SNU_INTRA_** |
| --- | --- | --- | --- | --- |
| **ESCoT**  **(r, p_FDR_)** | 0.586,  <0.001 | 0.777,  <0.001 | 0.499,  <0.001 | 0.544,  <0.001 |
| **ToM_C_**  **(rho, p_FDR_)** | - | 0.287,  0.016 | 0.156,  0.212 | 0.201,  0.120 |
| **ToM_A_**  **(r, p_FDR_)** | - | - | 0.125,  0.231 | 0.129,  0.231 |
| **SNU_INTER_**  **(r, p_FDR_)** | - | - | - | 0.197,  0.120 |

**Table S2.2. Partial correlations between conventional social cognition measures. r Pearson correlation coefficient was reported; rho Spearman correlation coefficient was reported; p-values were adjusted for FDR correction. ESCoT, Edinburgh Social COgnition Test; ToM_C_, cognitive theory of mind subscore; ToM_A_, affective theory of mind subscore; SNUI_NTER_, interpersonal social norm understanding subscore; SNU_INTRA_, intrapersonal social norm understanding subscore.**

**S3.** **Predictors on social cognition tests.**

Concerning predictors of RME, models did not differ from a null model (Table S3).

|  | **Predictors on RME** | **β** | **S.E.** | **t** | ***p-value*** | ***F*** | ***Omnibus***  ***p-value*** |
| --- | --- | --- | --- | --- | --- | --- | --- |
| Step 1 | Age | -0.82 | 0.90 | -0.92 | 0.363 | 1.52 | 0.202 |
|  | Years of education | 0.13 | 0.13 | 1.00 | 0.319 |  |  |
|  | Gender | 1.30 | 0.72 | 1.81 | 0.074 |  |  |
|  | Participation before/during pandemic (covariate) | 1.37 | 0.89 | 1.54 | 0.126 |  |  |
|  | Intercept | 23.53 | 2.15 | 10.96 | <0.001 |  |  |
| Step 2 | Age | -0.39 | 1.19 | -0.33 | 0.746 | 1.96 | 0.054 |
|  | Years of education | -0.10 | 0.15 | -0.65 | 0.520 |  |  |
|  | Gender | **1.53** | **0.74** | **2.07** | **0.042** |  |  |
|  | Digit_Forward | -0.11 | 0.34 | -0.34 | 0.737 |  |  |
|  | Digit_Backward | 0.57 | 0.37 | 1.57 | 0.120 |  |  |
|  | Stroop_Time | 0.06 | 0.06 | 1.02 | 0.312 |  |  |
|  | VCI | **0.12** | **0.04** | **2.90** | **0.005** |  |  |
|  | PRI | -0.02 | 0.03 | -0.66 | 0.509 |  |  |
|  | Participation before/during pandemic (covariate) | 2.16 | 1.04 | 2.07 | 0.041 |  |  |
|  | Intercept | 12.37 | 4.43 | 2.79 | 0.007 |  |  |

Table S3- Multiple regression results of predictors on Reading the Mind in the Eyes Test total score. The period of research participation (before or during the pandemic) was inserted in the model as a covariate.

Exploring predictors of SNQ total score, the model comparisons showed no significant difference. Both models showed age and gender as significant predictors on SNQ (Model 1: R^2^ = 0.200; Model 2: R^2^ = 0.229) (Table S4).

|  | **Predictors on SNQ** | **β** | **S.E.** | **t** | ***p-value*** | ***F*** | ***Omnibus***  ***p-value*** |
| --- | --- | --- | --- | --- | --- | --- | --- |
| Step 1 | Age | **-1.20** | **0.44** | **-2.76** | **0.007** | 5.50 | <0.001 |
|  | Years of education | -0.00 | 0.06 | -0.07 | 0.946 |  |  |
|  | Gender | **1.13** | **0.35** | **3.25** | **0.002** |  |  |
|  | Participation before/during pandemic (covariate) | -0.13 | 0.43 | -0.29 | 0.771 |  |  |
|  | Intercept | 18.39 | 1.04 | 17.60 | <0.001 |  |  |
| Step 2 | Age | **-1.17** | **0.60** | **-1.95** | **0.055** | 2.74 | 0.008 |
|  | Years of education | -0.06 | 0.08 | -0.78 | 0.440 |  |  |
|  | Gender | **1.22** | **0.38** | **3.24** | **0.002** |  |  |
|  | Digit_Forward | -0.04 | 0.17 | -0.24 | 0.813 |  |  |
|  | Digit_Backward | 0.05 | 0.19 | 0.28 | 0.783 |  |  |
|  | Stroop_Time | 0.01 | 0.03 | 0.29 | 0.775 |  |  |
|  | VCI | 0.01 | 0.02 | 0.45 | 0.656 |  |  |
|  | PRI | 0.02 | 0.01 | 1.32 | 0.192 |  |  |
|  | Participation before/during pandemic (covariate) | -0.14 | 0.53 | -0.27 | 0.786 |  |  |
|  | Intercept | 16.14 | 2.26 | 7.16 | <0.001 |  |  |

Table S4- Multiple regression results of predictors on SNQ total score. The period of research participation (before or during the pandemic) was inserted in the model as a covariate.

Regarding SS, the model comparisons did not differ from a null model (Table S5).

|  | **Predictors on SS** | **β** | **S.E.** | **t** | ***p-value*** | ***F*** | ***Omnibus***  ***p-value*** |
| --- | --- | --- | --- | --- | --- | --- | --- |
| Step 1 | Age | **-0.90** | **0.43** | **-2.11** | **0.038** | 1.34 | 0.260 |
|  | Years of education | 0.02 | 0.06 | 0.34 | 0.736 |  |  |
|  | Gender | -0.17 | 0.34 | -0.51 | 0.615 |  |  |
|  | Participation before/during pandemic (covariate) | 0.75 | 0.42 | 1.76 | 0.081 |  |  |
|  | Intercept | 12.50 | 1.02 | 12.23 | <0.001 |  |  |
| Step 2 | Age | -0.78 | 0.59 | -1.32 | 0.191 | 0.96 | 0.479 |
|  | Years of education | -0.01 | 0.08 | -0.12 | 0.909 |  |  |
|  | Gender | -0.16 | 0.37 | -0.44 | 0.662 |  |  |
|  | Digit_Forward | -0.12 | 0.17 | -0.73 | 0.467 |  |  |
|  | Digit_Backward | 0.31 | 0.18 | 1.68 | 0.096 |  |  |
|  | Stroop_Time | 0.01 | 0.03 | 0.21 | 0.834 |  |  |
|  | VCI | 0.00 | 0.02 | 0.19 | 0.849 |  |  |
|  | PRI | 0.00 | 0.01 | 0.41 | 0.686 |  |  |
|  | Participation before/during pandemic (covariate) | 0.49 | 0.52 | 0.95 | 0.342 |  |  |
|  | Intercept | 11.26 | 2.20 | 5.11 | <0.001 |  |  |

Table S5- Multiple regression results of predictors on the Strange Stories total score. The period of research participation (before or during the pandemic) was inserted in the model as a covariate.

Concerning predictors on the Yoni test, the model comparisons showed no significant difference. Both Model 1 (R^2^ = 0.199) and Model 2 (R^2^ = 0.204) report age as the only significant predictor on Yoni performance (Table S6).

|  | **Predictors on Yoni** | **β** | **S.E.** | **t** | ***p-value*** | ***F*** | ***Omnibus***  ***p-value*** |
| --- | --- | --- | --- | --- | --- | --- | --- |
| Step 1 | Age | **-12.24** | **3.02** | **-4.06** | **<0.001** | 5.46 | <0.001 |
|  | Years of education | 0.20 | 0.45 | 0.44 | 0.662 |  |  |
|  | Gender | 3.11 | 2.42 | 1.29 | 0.201 |  |  |
|  | Participation before/during pandemic (covariate) | 1.24 | 3.00 | 0.41 | 0.681 |  |  |
|  | Intercept | 70.73 | 7.24 | 9.77 | <0.001 |  |  |
| Step 2 | Age | **-12.68** | **4.24** | **-2.99** | **0.004** | 2.36 | 0.020 |
|  | Years of education | 0.07 | 0.54 | 0.14 | 0.890 |  |  |
|  | Gender | 3.65 | 2.66 | 1.37 | 0.173 |  |  |
|  | Digit_Forward | 0.72 | 1.22 | 0.59 | 0.558 |  |  |
|  | Digit_Backward | -0.29 | 1.31 | -0.22 | 0.827 |  |  |
|  | Stroop_Time | -0.01 | 0.22 | 0.04 | 0.970 |  |  |
|  | VCI | 0.07 | 0.15 | 0.44 | 0.663 |  |  |
|  | PRI | -0.01 | 0.10 | -0.16 | 0.872 |  |  |
|  | Participation before/during pandemic (covariate) | 2.08 | 3.73 | 0.56 | 0.578 |  |  |
|  | Intercept | 63.76 | 15.87 | 4.02 | <0.001 |  |  |

Table S6- Multiple regression results of predictors on the Yoni total score. The period of research participation (before or during the pandemic) was inserted in the model as a covariate.

Regarding AQ, the model comparisons did not differ from a null model (Table S7).

|  | **Predictors on AQ** | **β** | **S.E.** | **t** | ***p-value*** | ***F*** | ***Omnibus***  ***p-value*** |
| --- | --- | --- | --- | --- | --- | --- | --- |
| Step 1 | Age | 0.04 | 0.05 | 0.82 | 0.412 | 1.49 | 0.211 |
|  | Years of education | 0.33 | 0.24 | 1.35 | 0.181 |  |  |
|  | Gender | -1.96 | 1.30 | -1.51 | 0.136 |  |  |
|  | Participation before/during pandemic (covariate) | -0.74 | 1.60 | -0.46 | 0.643 |  |  |
|  | Intercept | 11.29 | 3.84 | 2.94 | 0.004 |  |  |
| Step 2 | Age | 0.08 | 0.07 | 1.16 | 0.249 | 1.00 | 0.450 |
|  | Years of education | 0.34 | 0.29 | 1.15 | 0.251 |  |  |
|  | Gender | -1.87 | 1.40 | -1.34 | 0.184 |  |  |
|  | Digit_Forward | 0.44 | 0.64 | 0.69 | 0.494 |  |  |
|  | Digit_Backward | -0.02 | 0.69 | -0.03 | 0.977 |  |  |
|  | Stroop_Time | 0.09 | 0.12 | 0.72 | 0.474 |  |  |
|  | VCI | -0.11 | 0.08 | -1.31 | 0.192 |  |  |
|  | PRI | 0.05 | 0.05 | 0.977 | 0.331 |  |  |
|  | Participation before/during pandemic (covariate) | -2.24 | 1.97 | -1.13 | 0.260 |  |  |
|  | Intercept | 11.76 | 8.72 | 1.35 | 0.181 |  |  |

Table S7- Multiple regression results of predictors on the AQ. The period of research participation (before or during the pandemic) was inserted in the model as a covariate.

Also regarding EQ, the model comparisons did not differ from a null model (Table S8).

|  | **Predictors on AQ** | **β** | **S.E.** | **t** | ***p-value*** | ***F*** | ***Omnibus***  ***p-value*** |
| --- | --- | --- | --- | --- | --- | --- | --- |
| Step 1 | Age | 0.00 | 0.07 | 0.02 | 0.982 | 1.74 | 0.147 |
|  | Years of education | -0.01 | 0.33 | -0.03 | 0.975 |  |  |
|  | Gender | 4.61 | 1.78 | 2.59 | 0.011 |  |  |
|  | Participation before/during pandemic (covariate) | 0.71 | 2.19 | 0.32 | 0.745 |  |  |
|  | Intercept | 44.05 | 5.23 | 8.41 | <0.001 |  |  |
| Step 2 | Age | -0.00 | 0.09 | -0.10 | 0.921 | 1.01 | 0.439 |
|  | Years of education | -0.15 | 0.40 | -0.38 | 0.704 |  |  |
|  | Gender | 5.24 | 1.91 | 2.74 | 0.008 |  |  |
|  | Digit_Forward | 0.89 | 0.88 | 1.01 | 0.317 |  |  |
|  | Digit_Backward | -1.00 | 0.95 | -1.05 | 0.294 |  |  |
|  | Stroop_Time | 0.02 | 0.16 | 0.10 | 0.917 |  |  |
|  | VCI | 0.03 | 0.11 | 0.26 | 0.792 |  |  |
|  | PRI | 0.04 | 0.07 | 0.59 | 0.555 |  |  |
|  | Participation before/during pandemic (covariate) | 1.50 | 2.70 | 0.56 | 0.579 |  |  |
|  | Intercept | 37.53 | 11.94 | 3.14 | 0.002 |  |  |

Table S8- Multiple regression results of predictors on the EQ. The period of research participation (before or during the pandemic) was inserted in the model as a covariate.

**References**

Weintraub, S., Besser, L., Dodge, H.H., Teylan, M., Ferris, S., Goldstein, F.C., Giordani, B., Kramer, J., Loewenstein, D., Marson, D., Mungas, D., Salmon, D., Welsh-Bohmer, K., Zhou, X.H., Shirk, S.D., Atri, A., Kukull, W.A., Phelps, C., & Morris, J. C. (2018), «Version 3 of the Alzheimer Disease Centers' Neuropsychological Test Battery in the Uniform Data Set (UDS)». Alzheimer disease and associated disorders, 32(1), pp. 10–17.
